# Neurocognitive outcomes after treatment of unruptured anterior communicating artery aneurysms – a systematic review

**DOI:** 10.1007/s10143-026-04423-6

**Published:** 2026-07-31

**Authors:** Jorn P. Van Der Veken, Christopher D. Ovenden, Vanesa M. Tomatis, Felix Paterson, Vincent Oxenham, Candice Delcourt, Marcus A. Stoodley

**Affiliations:** 1 Department of Neurosurgery, Flinders Medical Centre, Bedford Park, Australia; 2 Department of Neurosurgery, Royal Adelaide Hospital, Adelaide, Australia; 3 Department of Clinical Medicine, Faculty of Medicine, Health and Human Sciences, Macquarie University, Sydney, Australia; 4 Adelaide Medical School, Faculty of Health and Medical Sciences, University of Adelaide, Adelaide, Australia; 5 South Australia Medical Imaging, Flinders Medical Centre, Bedford Park, Australia; 6 School of Psychological Sciences, Faculty of Medicine, Health and Human Sciences, Macquarie University, Sydney, Australia; 7 Neurology Department, Royal North Shore Hospital, Sydney, Australia; 8 The George Institute for Global Health, Faculty of Medicine, University of New South Wales, Sydney, Australia

**Keywords:** Anterior communicating artery, Aneurysm, Cognition

## Abstract

Cognitive changes after treatment of ruptured anterior communicating artery (AComA) aneurysms have been recognized for decades. Whether such deficits occur after elective treatment of unruptured aneurysms remains uncertain. A systematic search in MEDLINE (via PubMed and via Ovid), Embase, Scopus, Web of Science, CENTRAL and PsycINFO was conducted from inception to 31 August 2025, in accordance with PRISMA guidelines. Duplicate records were removed prior to screening. Studies were eligible if they included adult patients undergoing elective treatment of at least 1 unruptured AComA aneurysm, reported pre- and post-treatment neurocognitive testing across at least one cognitive subdomain, and differentiated outcomes from other aneurysm locations. Critical appraisal was performed using the Newcastle–Ottawa Scale, and certainty of evidence was assessed using the GRADE framework. Eight studies comprising 95 patients were included. In most studies, patients underwent surgical clipping (*n* = 82), with a minority treated endovascularly (*n* = 13). In five out of eight studies, patients who underwent elective treatment of an AComA aneurysm had worse cognitive outcomes than patients with aneurysms at non-AComA locations. Neurocognitive deficits may be detected in a substantial proportion of patients undergoing elective treatment of unruptured AComA aneurysms when sensitive neuropsychological assessment is applied. However, the current evidence base is limited by small sample sizes, heterogeneity in neuropsychological assessment, and a predominance of surgically treated cases.

**Supplementary Information:**

The online version contains supplementary material available at 10.1007/s10143-026-04423-6.

## Introduction

Intracranial aneurysms are estimated to occur in approximately 3.2% of the general population [1]. Management decisions are based on a careful assessment of the risk associated with intervention versus the natural history of the aneurysm (rupture risk). With the advent of safer endovascular devices and less invasive surgical techniques, the threshold for treating unruptured aneurysms has lowered, leading to an increase in the number of patients undergoing treatment [2, 3]. 

When counselling patients on procedural risks, whether surgical or endovascular, clinicians generally quantify the risk of major stroke, permanent neurological deficit, or mortality [4, 5]. However, predicting the likelihood of more subtle complications, particularly those affecting learning and memory, visuospatial ability and various aspects of executive function such as verbal fluency, divided-attention, cognitive flexibility, multitasking, problem solving and planning, are more challenging. Additionally, emotional regulation and foundational skills such as attention, working memory and information processing abilities and speed can also be impacted. For many patients, impairments in these domains have a substantial impact on self-care, social and professional functioning, and quality of life. These deficits will not be captured by routine outcome scales such as the modified Rankin Scale (mRS) or Glasgow Outcome Scale (GOS) [6]. 

Approximately 30–40% of intracranial aneurysms arise from the anterior communicating artery (AComA) [7]. The AComA lies beneath the frontal lobes and provides vascular supply predominantly to the mesiofrontal, frontobasal, prefrontal and subcallosal regions, areas critically involved in cognition and behaviour [8]. It is well accepted that treatment of ruptured AComA aneurysms can be associated with neurocognitive and psychological sequelae [9]. These tend to be less severe in patients receiving endovascular treatment compared to those who had surgical clipping [10]. However, it remains unclear whether neurocognitive deficits occur following elective treatment of unruptured AComA aneurysms. To address this question, we conducted a systematic review of the literature focusing on studies that report short- and long-term neurocognitive outcomes following elective AComA aneurysm treatment.

## Methods

### Search strategy

We conducted a literature review in accordance with the 2020 Preferred Reporting Items of Systematic Reviews and Meta-Analyses (PRISMA) statement [11]. MEDLINE (via PubMed and via Ovid), Embase, Scopus, Web of Science, CENTRAL and PsycINFO were searched from inception until the 31 st of August 2025.

**Fig. 1:**
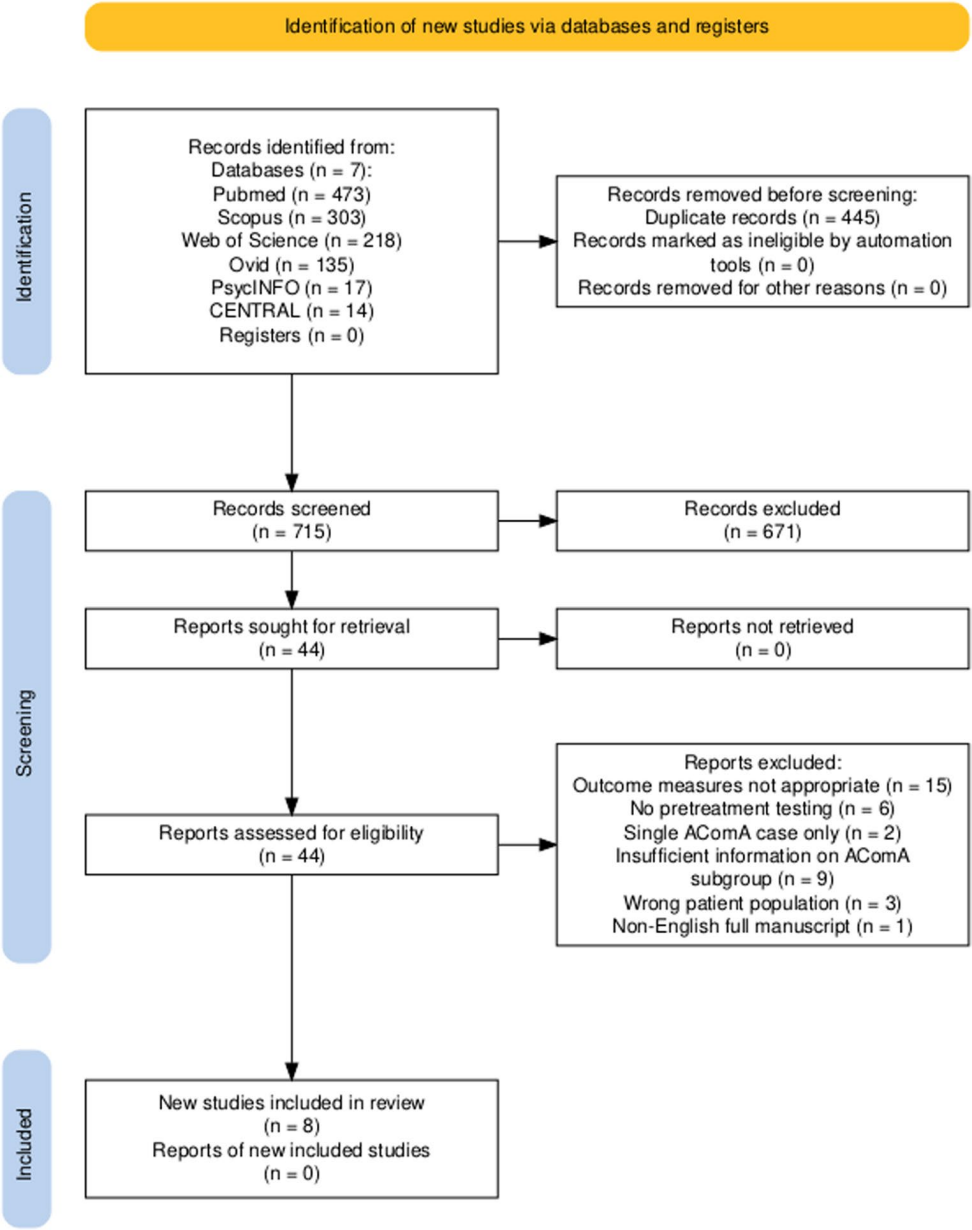
PRISMA 2020 flow diagram of study selection

Titles and abstracts were independently screened by the first two authors to identify potentially eligible studies. Full-text articles were retrieved and assessed for inclusion when studies met eligibility criteria or when relevance could not be determined from the abstract alone. Disagreements were resolved by consensus. The study selection process is illustrated in Fig. 1. PROSPERO registration: CRD420251234088. The full electronic search strategies for all databases are provided in Supplementary Material.

### Eligibility criteria

This systematic review included studies that involved adult patients. Articles were limited to the English language. Only studies that included elective treatment of more than one patient with an unruptured AComA aneurysm were included. In studies that included patients with multiple intracranial aneurysms, only patients undergoing treatment of a single AComA aneurysm were included. Patients undergoing simultaneous treatment of multiple aneurysms were excluded. We excluded studies where no pretreatment cognitive assessment was performed. Studies not differentiating between ruptured and unruptured aneurysms were excluded. Studies that assessed cognition exclusively using a single screening tool, such as the Mini-Mental State Examination (MMSE) or the Montreal Cognitive Assessment (MoCA), or that reported solely functional outcomes (e.g., Glasgow Outcome Scale (GOS) or modified Rankin Scale (mRS)), were excluded, as these instruments are insufficient to detect deficits within specific cognitive subdomains. Only studies that evaluated at least one defined cognitive subdomain - such as attention, working memory, processing speed, verbal or non-verbal learning and memory, visuospatial ability, or executive functions (including verbal fluency, cognitive flexibility, and planning), were eligible for inclusion.

### Data extraction

Abstracts and full text were independently reviewed by two authors (JVDV and CO) with discrepancies settled by consensus. Data extraction occurred in a pre-specified manner with collected data including author information, year of publication, mode of treatment, timing, type and outcome of neurocognitive assessment and surgical technique. When aneurysm location was ambiguously reported as ACA, authors were contacted to clarify AComA involvement.

### Data synthesis

A meta-analysis was not performed due to the limited number of studies with comparable outcome data and substantial clinical and methodological heterogeneity, including differences in intervention, neuropsychological assessment tools, timing of evaluation, and outcome reporting formats, precluding meaningful quantitative pooling. A narrative synthesis was therefore undertaken.

### Quality assessment

Two reviewers (JVDV and CO) used the Newcastle-Ottawa Scale (NOS) to assess the quality of the non-randomised studies included in this systematic review (see Appendix 1). Certainty of evidence for cognitive outcomes was evaluated using the GRADE framework. (see Appendix 2)

## Results

From the literature search 715 articles were screened after duplicates had been removed.

Following the eligibility check, a total of 44 articles were screened: 36 articles were excluded, and 8 articles were included for final review. These 8 articles describe 95 AComA patients in total: 82 treated surgically, and 13 by endovascular means. There was substantial heterogeneity in both neuropsychological tests and timing of assessment. (Table 1)

Characteristics of the included studies are summarized in Table 1.

**Table 1:** Characteristics of studies included in the systematic review evaluating neurocognitive outcomes following elective treatment of unruptured anterior communicating artery aneurysms

Author and year	Unruptured AComA aneurysm/ Non-AComA aneurysms	Treatment Surgical/Endovascular (AComA only)	Study design	Cognitive tests used	Timing of testing	Surgical technique	Results
1999 Fukunaga [12]	8/19^a^	8/0	Prospective	1) MMSE 2) Kana-Hiroi 3) Maze test	Preop 1 month postop 3 months postop	Pterional and interhemispheric. No gyrus rectus aspiration.	**7/8 patients with an AComA aneurysm deteriorated on the Kana Hiroi test (temporary**,** recovered by 3 months postop)**
2003 Ohue [13]	7/28^b^	7/0	Prospective	1) MMSE 2) Kana-Hiroi 3) Kohs Block Design 4) Miyake’s Memory test	Preop 1 month postop 6 months postop	Pterional and interhemispheric. Gyrus rectus aspiration.	**Patients with an AComA aneurysm showed a significantly higher incidence of neuropsychological deterioration after surgery 5/7 (71%) compared to 4/10 MCA and 1/16 ICA (p < 0.01)** Systemic diseases and an interhemispheric approach are also associated with worse cognitive outcomes. Kohs’ Block Design test 1 month after surgery showed full recovery 6 months later. On the other hand, only 67% and 40% of the patients with neuropsychological deterioration in Kana-Hiroi and Miyake’s Memory tests 1 month after surgery showed full recovery 6 months later
2013 Pereira-Filho [14]	2/33^c^	2/0	Prospective	1) MMSE 2) WAIS-III 3) WMS-III 4) BNT 5) CWST 6) VFT 7) FrSBe	Preop 3 months postop 3 years postop	Pterional. Retractors used intermittently.	**The 2 patients with AComA aneurysms did not perform significantly differently on the cognitive and behavioural tests when compared to the patients with aneurysms in other topographies**
2014 Inoue [15]	19/109^d^	19/0	Retrospective	1) WAIS-III (VIQ and PIQ) 2) WMS-memory 3) WMS-attention	Preop (1 week) 6 months postop	Interhemispheric Neuromonitoring	**Patients with AComA aneurysms in the present study did not show reduced neurocognitive function (composite score) when compared with patients with aneurysms in other locations** structural damage, as visualized by T2-weighted imaging, at 6 months resulting from surgical manipulation can cause subtle but significant adverse effects on postoperative neurocognitive function
2014 Shibahashi [16]	17/54	17/0	Prospective	1) MMSE 2) FAB 3) TMT-A 4) TMT-B 5) RCPM	Preop (1 week) within 12 days postop	Pterional (8/17) and interhemispheric (9/17).	**Deterioration was observed after surgery in the TMT-A score in elderly patients P = 0.049) and in patients with large aneurysms (P = 0.042), AComA aneurysms (P = 0.016), and long operation durations**. Deterioration of the TMT-A score in the AComA aneurysm group was evident but only in interhemispheric approach cases (*P* = 0.009)
2016 Chung [17]	15/51	11/4	Prospective	1) ACCPT 2) CWST 3) VLT	Preop (2–5 days) 6 months postop	Pterional minicraniotomy	**AComA aneurysms were associated with decreased WCT scores after 6 months compared to aneurysms on the MCA p = 0.032)**
2019 Caveney [18]	17/68	14/3	Prospective	1) RAVLT (Trial 1 and total) 2) ROCFT 3) CWST 4) GP Dom H & Non-Dom H 5) TMT-A 6) TMT-B 7) Dig Symb 8) COWA	Preop 2 weeks postop 3 months postop 6 months postop 12 months postop	Not specified.	**Patients with an AComA aneurysm had worse postoperative results on executive functioning and memory**,** compared to non-AComA aneurysm patients**,** independent of procedure**
2025 Kanazawa [19]	10/47	4/6	Retrospective	1) MMSE 2) FAB 3) S-PA1 4) S-PA2	Preop 6 m postop 2 years postop	Pterional (3/4) Interhemispheric (1/4)	**A decrease in cerebral blood flow on 123IMP SPECT was seen in both endovascular and surgical groups (more in the surgical cohiort)**,** but no cognitive changes were seen**

*ACCPT* auditory controlled continuous performance test, *BNT* Boston naming test, *COWA* controlled oral word association test, *CWST* coloured word stroop test, *Dig Symb* digit symbol substitution test (part of WAIS), *FAB* frontal assessment battery, *FrSBe* frontal systems behavior scale, *GP Dom and Non-Dom* grooved pegboard dominant and non-dominant hand, *MMSE* mini mental state examination, *RAVLT* rey auditory verbal learning test, *RCPM* raven’s colored progressive matrices, *ROCFT* rey osterrieth complex figure test, *S-PA1* standard verbal paired-associate learning test (related words), *SPA2* standard verbal paired-associate learning test (unrelated words), *TMT* trail making test, *VFT* verbal fluency test, *VLT* verbal learning test, *WAIS* wechsler adult intelligence scale III

Legend: a: additional 3 pcferent sites; b: additional 8 patients had multiple aneurysms at different sites treated simultaneously; c: additional 5 patients had multiple aneurysms at different sites treated simultaneously

Across the included studies, postoperative cognitive changes were most commonly reported in three domains: executive function and attention (TMT-A/B, Stroop/WCT, ACCPT), verbal learning and memory (Kana-Hiroi, Miyake, VLT, RAVLT), whereas visuospatial functions demonstrated more mixed or transient changes (Kohs Block Design, ROCFT).

Overall methodological quality was moderate to good across included studies, with Newcastle–Ottawa Scale scores ranging from 4 to 9. (Appendix 1).

## Discussion

### Summary and interpretation of findings

This systematic review identified only eight studies encompassing 95 patients with unruptured anterior communicating artery (AComA) aneurysms, underscoring the remarkable paucity of data on neurocognitive outcomes despite the frequency of elective treatment worldwide.

Despite the small number of included studies, five out of eight reported a higher incidence of neurocognitive deficits after elective AComA aneurysm treatment compared to other locations (Table 1). These cognitive changes most commonly involved executive function and attention, as well as verbal learning and memory. However, not all studies demonstrated significant differences, which may reflect heterogeneity in neuropsychological testing methods, timing of postoperative assessment, and the relatively small sample sizes of the included cohorts.

### Gaps and challenges in the current evidence

Although an abundance of literature has been published looking at neurological outcomes of treated aneurysms, few use an outcome parameter adequately adapted to the aneurysm location [20–29]. For elective AComA aneurysms, cognitive, behavioral, and psychological outcomes due to locoregional injuries are often not picked up with routine outcome scales such as the modified Rankin Scale (mRS) or Glasgow Outcome Scale (GOS) [6]. Other outcome scales such as MMSE and MOCA are most commonly used to screen for global cognitive function and were designed specifically to pick up cognitive changes typically observed within dementia cohorts. As these tools were not designed to detect subtle, domain-specific deficits, they likely underestimate clinically meaningful cognitive changes in this population. Therefore, the cognitive changes noted in these studies and in the AComA population may be underrepresented. To date, no consensus neuropsychological assessment framework exists for evaluating cognitive outcomes following treatment of unruptured AComA aneurysms.

Other studies lacked pre-treatment assessment or combined ruptured and unruptured cohorts [17, 26, 30–37]. 

The majority of included patients underwent surgical clipping. This highlights a particular gap in knowledge on neurocognitive outcomes after endovascularly treated aneurysms. DWI hits are frequently seen after endovascular treatment of aneurysms, but how this affects neurocognitive outcomes, particularly in AComA aneurysms, is currently unknown [38, 39]. 

Surgical technique was described in 7 out of 8 cases, and varied between interhemispheric and pterional, also the use of retractors and neuromonitoring varied. Interestingly, Ohue et al. concluded that an interhemispheric approach increased the risk of postoperative cognitive deficits, whereas all the patients in Inoue et al. series were operated via an interhemispheric approach and did not show any worse postoperative outcomes [13, 15]. This might reflect the difference in methodology or the sensitivities of the tests used.

Procedural risks and potential complications vary substantially by aneurysm location; however, many studies do not differentiate between anatomical locations and assess unruptured aneurysms as a single group [29, 32, 34, 40]. 

Despite these limitations, the available data collectively suggest that neurocognitive morbidity is not uncommon after technically successful AComA aneurysm repair. This can be explained by the unique anatomical characteristics of the AComA complex, where even small ischemic or mechanical insults can disrupt critical frontobasal and mesiofrontal connections. Understanding these anatomical relationships is therefore essential to interpret the mechanisms underlying the observed cognitive sequelae.

### Anatomical mechanisms underlying cognitive vulnerability

Many papers have been dedicated to describing the variations that occur in the AComA complex [41–44]. From a surgical perspective, some of the varying anatomical factors that are taken into account when deciding on the appropriate surgical approach are: A1 dominance, direction of the dome, distance of the AComA from the planum sphenoidale, and anteroposterior relationship of the A2s. Intraoperatively, multiple branches need to be identified in the small working corridor: both A1s and A2s, the recurrent artery of Heubner, the medial orbitofrontal arteries, and the frontopolar arteries. Adding to the complexity is the variation in size and angulation of the AComA; not uncommonly, a duplication, hypoplasia, or fenestration is present. Further variations exist in the number and location of perforating branch vessels arising from the posterior, superior, and inferior walls [43]. 

The perforating branches of the AComA perfuse the hypothalamus, optic chiasm, septal area (subcallosal area and paraterminal gyrus), anterior commissure, column of the fornix as well as the septum pellucidum, rostrum and genu of the corpus callosum, and anterior cingulate gyrus [45, 46]. The number of perforators ranges from 0 to 11 and can be grouped into the hypothalamic, chiasmatic and subcallosal or median callosal arteries [22, 39, 41]. 

These perforators are at risk of occlusion during both surgery and endovascular treatment. Indirect evidence suggests that injury to these vessels may contribute to neurocognitive deficits following treatment of AComA aneurysms. In particular, infarction in the territory of the subcallosal artery has been associated with postoperative cognitive impairment [47, 48]. Mugikura et al. performed 3D MRI imaging in 10 consecutive patients with postoperative amnesia. Eight patients had a ruptured AComA aneurysm, 2 had undergone elective treatment. All 10 patients had infarcted foci in the territory of the subcallosal artery (the column of the fornix, anterior commissure, and paraterminal gyrus), and 9 patients had bilateral foci [47]. Another indirect argument suggesting AComA perforators as the cause for postoperative cognitive deficits is the increased incidence when AComA aneurysms are trapped [47, 48]. However, none of the studies included in this review systematically correlated postoperative imaging findings with neurocognitive outcomes, representing an important area for future research.

### Treatment modality and potential mechanisms of cognitive injury

Treatment modality may influence the mechanism and pattern of injury. In microsurgical clipping, perforators may remain in the surgeon’s blind spot, depending on the aneurysm size and dome projection [49]. This is particularly relevant in a pterional approach, and interhemispheric approaches have been advocated to improve visualization of these vessels [21]. More recently, endoscopy has been recommended to aid in clipping an aneurysm, as it allows inspection of hidden corners, thereby reducing the risk of trapped perforators [50, 51]. When treating AComA aneurysms surgically, the use of retractors has also been proven to be a source of brain damage [52]. Gyrus rectus resection, frequently performed to enhance exposure, may also contribute to neurocognitive deficits given its proximity to the limbic system [53]. 

These small perforators are also at risk during endovascular treatment because they are difficult to visualize [54]. However, the pattern of brain injury tends to differ after endovascular treatment. A prospective trial compared post-treatment lesions evident on Diffusion Weighted Imaging (DWI) between a microsurgical and an endovascular cohort; post-therapeutic lesions were detected in both groups with similar frequencies but with different patterns [38]. In the endovascular setting, this is typically characterized by a diffuse shower of small, spatially distributed microembolic DWI lesions rather than focal territorial infarction. Meta-analytic data suggest that new DWI lesions are present in approximately 50% of patients following endovascular aneurysm treatment, increasing to 67% with flow diversion [55]. 

### Possible other factors influencing the cognitive outcomes of elective AComA aneurysm treatment

A consistent discrepancy exists between the high incidence of radiological injury detected after aneurysm treatment and the relatively low rates of clinically apparent neurological deficit reported in outcome studies [24, 39, 56]. This can be partially explained by the limited sensitivity of conventional outcome measures, but also by the intrinsic capacities of the brain to tolerate varying degrees of neurological injury. Brain reserve refers to the discrepancy between the extent of structural brain damage present and the clinical symptoms observed; individuals with greater brain reserve can sustain more injury before showing deficits. Brain resilience, by contrast, reflects the brain’s ability to compensate for, adapt to, or reorganize in response to damage once it has occurred. These concepts are particularly relevant in cerebrovascular disease, where they help explain ‘silent brain infarcts’ or a variation in the severity of vascular cognitive impairment (VCI) and post-stroke cognitive impairment (PSCI) despite similar lesion burdens [57]. 

Cognitive reserve and cognitive resilience extend these ideas to the functional domain of cognition. Cognitive reserve refers to an individual’s capacity to maintain cognitive performance despite brain ageing, pathology, or injury. It is shaped by lifelong factors such as education, occupational complexity, leisure activities, and genetic influences. Cognitive resilience emphasizes the ability not only to maintain but also to recover cognitive functioning following neurological insult, highlighting the dynamic adaptability of cognitive networks [58]. 

The few studies that have examined the cognitive outcomes of treated AComA aneurysms suggest that several other potential patient and treatment factors may influence outcomes, including the patient’s age, comorbidities, and previous cognitive decline, all of which can impact brain vulnerability or reserve. The size and site of the craniotomy, the approach (pterional versus interhemispheric), and the length of the procedure have also been suggested to influence cognitive outcomes [13, 17]. Hemispheric dominance may influence neurocognitive outcomes, particularly for language and memory. However, most studies did not report laterality or account for dominance, limiting further analysis. Additionally, the use of insensitive and less specific cognitive screening measures or a failure to consider practice effect likely contributes to variance and possible underreporting of cognitive changes following AComA aneurysm treatment. As stable postoperative scores may still represent a relative decline when compared with the improvement expected from repeated testing, neurocognitive deficits may be underestimated.

### Limitations of this systematic review

There are some obvious limitations with this systematic review. Although the included studies were generally of moderate methodological quality, the overall certainty of evidence for cognitive outcomes was rated as very low using the GRADE framework. This reflects the observational nature of the data, small sample sizes, heterogeneity of neuropsychological assessments, and imprecision of effect estimates. Furthermore, AComA aneurysms were frequently analyzed as part of larger, heterogeneous aneurysm cohorts, and detailed subgroup information on patient demographics, aneurysm characteristics, and surgical techniques was often not reported. Most included studies compared patients with AComA aneurysms to patients harboring aneurysms at other locations. Although this may help identify cognitive outcomes specific to the AComA region, patients with untreated aneurysms or matched healthy controls would better distinguish treatment-related cognitive changes from those associated with the presence of an intracranial aneurysm itself.

Finally, the predominance of surgically treated aneurysms limits conclusions regarding modality-specific cognitive outcomes, particularly in contemporary practice where many AComA aneurysms are managed endovascularly. In addition heterogeneity in cognitive testing and timing precluded quantitative synthesis.

## Conclusion

The available literature suggests that neurocognitive deficits may be detected in a substantial proportion of patients following elective treatment of unruptured AComA aneurysms when sensitive neuropsychological testing is performed. However, the current evidence base is limited by small sample sizes, methodological heterogeneity and an overall very low certainty of evidence. Well-designed prospective studies incorporating standardized neurocognitive assessments are needed to better define the incidence, severity, and longitudinal course of these deficits and to compare outcomes between surgical and endovascular treatment.

## Supplementary Information

Below is the link to the electronic supplementary material.

Supplementary Material 1 (XLSX 12.1 KB)

Supplementary Material 2 (DOCX 26.9 KB)

## Data Availability

No datasets were generated or analysed during the current study.

## Code availability

Nothing to disclose.
