# Supplementary material for: Neurocognitive outcomes after treatment of unruptured anterior communicating artery aneurysms – a systematic review
Source: Neurosurg Rev. 2026 Jul 31;49(1):500. doi: 10.1007/s10143-026-04423-6 (PMC13424562; doi:10.1007/s10143-026-04423-6)
Supplement: Supplementary file 2 — Supplementary Material 2 (DOCX 26.9 KB) [file 10143_2026_4423_MOESM2_ESM.docx]

Neurocognitive outcomes after elective treatment of unruptured anterior communicating artery aneurysms

| Outcome | No. of studies (design) | No. of participants | Risk of bias | Inconsistency | Indirectness | Imprecision | Publication bias | Overall certainty (GRADE) |
| --- | --- | --- | --- | --- | --- | --- | --- | --- |
| Post-treatment neurocognitive impairment | 8 (observational cohort studies) | 95 AComA patients | Serious | Serious | Serious | Serious | Undetected | ⨁◯◯◯ Very low |

Explanatory notes:

Risk of bias: Observational design, small samples, limited confounder adjustment.

Inconsistency: Heterogeneous cognitive domains, timing, and outcome reporting.

Indirectness: Variable test sensitivity and limited endovascular data.

Imprecision: Small AComA-specific samples and lack of precise effect estimates.
